# Favorable outcome of patients with lung adenocarcinoma harboring *POLE* mutations and expressing high *PD-L1*

**DOI:** 10.1186/s12943-018-0832-y

**Published:** 2018-04-12

**Authors:** Liang Liu, Jimmy Ruiz, Stacey S. O’Neill, Stefan C. Grant, W. Jeffrey Petty, Meng Yang, Kexin Chen, Umit Topaloglu, Boris Pasche, Wei Zhang

**Affiliations:** 1 Center for Cancer Genomics and Precision Oncology, Wake Forest Baptist Comprehensive Cancer Center, Wake Forest Baptist Medical Center, Winston Salem, NC 27157 USA; 2 Department of Cancer Biology, Winston Salem, NC 27157 USA; 3 Internal Medicine-Section of Hematology and Oncology, Winston Salem, NC 27157 USA; 4 Laboratory Medicine and Pathology, Wake Forest School of Medicine, Winston Salem, NC 27157 USA; 5 Department of Epidemiology and Biostatistics, National Clinical Research Center for Cancer, Key Laboratory of Cancer Prevention and Therapy of Tianjin, Tianjin Medical University Cancer Institute and Hospital, Tianjin, 300060 People’s Republic of China

**Keywords:** *POLE* mutation, *PD-L1* expression, Lung cancer adenocarcinoma, Lung cancer squamous cell carcinoma, Overall survival, Non-small cell lung cancer

## Abstract

Mutations in polymerase ε (*POLE*) confer favorable prognosis and outcomes in various cancer types, but their role in non-small cell lung cancer (NSCLC) is unknown. Utilizing the data of 513 patients with adenocarcinoma (LUAD) and 497 patients with squamous cell carcinoma (LUSC) from The Cancer Genome Atlas (TCGA) cohort, we tested the prognostic value of *POLE* mutations and programmed cell death ligand 1 (*PD-L1*) expression in the two main subtypes of NSCLC. *POLE* mutation is a favorable biomarker for the improved overall survival (OS) of the LUSC patients (*P =* 0.033, 28 mutant vs. 469 wildtype patients), but not that of the LUAD patients (*P =* 0.12, 31 mutant vs. 482 wildtype patients). *POLE*-mutant LUAD patients with high expression of *PD-L1* (Mut-High, *n* = 6) exhibited improved OS (*P =* 0.024) when compared to *POLE*-mutant patients with low *PD-L1* expression (Mut-Low, *n* = 24) and other patients without *POLE* mutation (*n* = 476). This benefit was not due to the high content of the tumor infiltrating lymphocytes. Instead, the antitumor immune response was activated in Mut-High patients so that these patients were likely responding more effectively to immuno-oncology (IO) treatments; whereas genes involved with metabolic pathways were enriched in Mut-Low group, which may cause the decreased OS of these patients. Our study sheds light on the molecular basis of NSCLC and adds to our understanding of responses to chemotherapy and IO therapy.

**Electronic supplementary material:**

The online version of this article (10.1186/s12943-018-0832-y) contains supplementary material, which is available to authorized users.

Non-small cell lung cancer (NSCLC) accounts for 85% of lung cancers and can be further broadly divided by histology into adenocarcinoma (LUAD), squamous cell carcinoma (LUSC), and large cell carcinoma. Therapies directed against molecular targets in NSCLC, including immuno-oncology (IO) treatments, have improved response rates and overall survival [1, 2]; however, predictive markers for response and patient outcomes are still lacking.

Polymerase ε (*POLE*) is a DNA polymerase involved in DNA replication and repair. *POLE* mutation is associated with an ultra-mutated phenotype and a good prognosis in uterine corpus endometrial carcinoma (UCEC) [3] and a subgroup of colorectal tumors (CRC) [4]. In NSCLC, the mutations of *POLE* and DNA mismatch repair (MMR) genes result in ultra-mutation in both LUAD and LUSC [5]. Interestingly, this event was also observed in patients who had favorable responses to immunotherapy [6]. Despite these observations, little is known about *POLE* mutations in NSCLC.

We analyzed the relationship of *POLE* mutations with programmed cell death ligand 1 (*PD-L1*) expression in patents with LUAD or LUSC in The Cancer Genome Atlas (TCGA) cohort. *PD-L1* expression was not a good prognostic predictor of patient outcomes for the two subtypes. *POLE* mutation alone could predict the overall survival (OS) for LUSC but not LUAD patients. Both *PD-L1* expression and tumor mutation burden (TMB) have exhibited associations with better response to immunotherapies in some but not all studies. Given that *POLE* mutations were correlated with high mutation rates, we hypothesized that the two predictors may jointly influence response to immunotherapy and survival outcomes. We found that the combination of *POLE* mutations and *PD-L1* expression was a favorable indicator for the improved OS of LUAD patients. Our analyses describe the molecular differences among the LUAD patients with *POLE* mutations and different levels of *PD-L1* expression and patients without *POLE* mutation, which may suggest distinct responses to chemotherapy and IO treatment.

## Results and discussion

### *POLE* mutation alone is a good prognostic biomarker for patients with lung squamous cell carcinoma but not lung adenocarcinoma

Analysis using genomic data across multiple types of cancers from the TCGA cohort (Additional file 1: Methods and Materials) showed that LUSC and LUAD are among the cancers with the most frequent *POLE* mutations (28/497 = 5.6% and 31/513 = 6.0%, respectively), which are close to the rates in UCEC (28/519 = 5.4%) and CRC cancers (32/594 = 5.4%). However, compared to the UCEC cancer patients whose mutations mostly locate in the proofreading domain, the mutations in NSCLC patients are distributed across the *POLE* gene body (Additional file 2: Figure S1A).

**Fig. 1:**
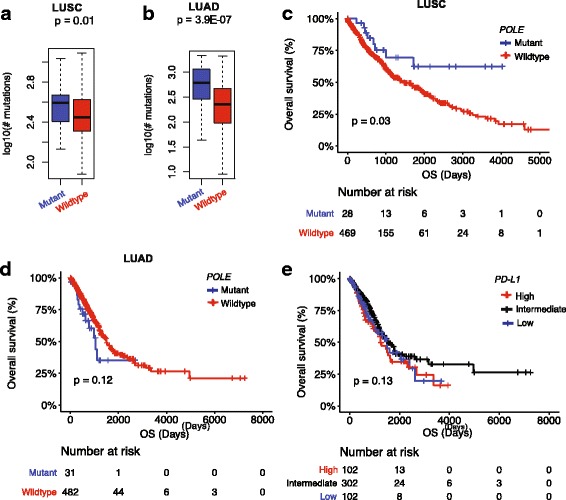
Characteristics of *POLE* mutations in the LUSC and LUAD cancers. **a** and (**b**) *POLE* mutations are associated with high tumor mutation burden (TMB) of the (**a**) LUSC and (**b**) LUAD patients; (**c**) and (**d**) as well as favorable outcomes of (**c**) LUSC patients but (**d**) not LUAD patients; (**e**) *PD-L1* expression cannot stratify LUAD patients

We tested whether *POLE* mutation has similar prognostic values in the LUSC and LUAD cancers. Analyses showed that *POLE*-mutant patients exhibited high mutational rates, compared with *POLE*-wild patients in both LUSC (*P =* 0.01) and LUAD (*P =* 3.9e-07) (Fig. 1a and b), consistent with previous findings [5]. Regarding patient outcomes, the *POLE*-mutant LUSC patients revealed improved OS (*P =* 0.03, Fig. 1c; see Additional file 3: Table S1 for descriptive characteristics). However, the presence of *POLE* mutations in LUAD patients (see Additional file 3: Table S2 for descriptive characteristics) were not associated with statistically significant improvement in OS (*P =* 0.12, Fig. 1d). The mortality rate of *POLE*-mutant LUAD patients was 96.8% (30/31 patients) at 5.5-year follow-up, which was higher than the 90.9% (438/482) of *POLE*-wild patients. Indeed, TMB was not associated with LUAD patient survival (*P =* 0.87, TMB-higher (top 20%, *n* = 103) vs. -low (bottom 20%, *n* = 101), Additional file 2: Figure S1B).

*PD-L1* expression level serves as a predictive biomarker to IO therapy response in a number of cancers types [7, 8], including advanced NSCLC [1, 2, 7]. Yet, analysis of TCGA samples with high- (top 20%, *n* = 102), low- (bottom 20%, *n* = 102), and intermediate (others, *n* = 302) *PD-L1* expression levels, who had not received IO therapy treatments, did not demonstrate that the LUAD patients with high *PD-L1* expression had longer OS (*P =* 0.13, Fig. 1e).

### LUAD patients with *POLE* mutations and *PD-L1* high expression level have the best survival

**Fig. 2:**
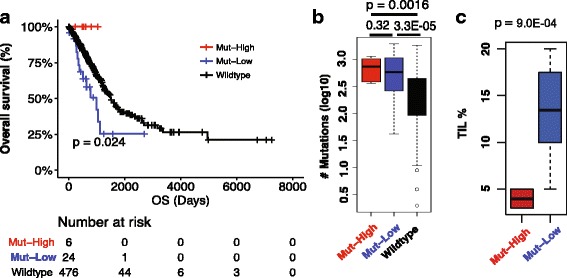
Characteristics of LUAD patients with both *POLE* mutations and high *PD-L1* expression. The combination of *POLE* mutation and high *PD-L1* expression is associated with (**a**) favorable LUAD patient survivals, (**b**) high TMB, and (**c**) low tumor infiltrating lymphocytes (TILs)

We categorized *POLE*-mutant LUAD patients into two groups based on their *PD-L1* expression levels: high (top 20%, Mut-High) and low (other 80%, Mut-Low)-*PD-L1* groups (Additional file 3: Table S3). Analysis showed that all the Mut-High patients (*n* = 6) survived. In contrast, Mut-Low LUAD patients (*n* = 24) had decreased OS, which was worse than patients with wildtype *POLE* (*n* = 476, *P =* 0.024, Fig. 2a). Mut-High patients had a similar mutation rate as compared to Mut-Low cases (*P =* 0.32), and both groups were significantly higher than *POLE*-wild patients (*P =* 0.0016 and 3.3e-05, respectively, Fig. 2b). It is worth noting that *PD-L1* expression could not stratify *POLE*-wild LUAD patients by OS (*P =* 0.55, 93 High (top 20%) vs. 383 Low (others), Additional file 2: Figure S2A) although high expression levels of *PD-L1* was associated with higher TMB (*P =* 0.0051, Additional file 2: Figure S2B).

We performed the same analyses in the LUSC cancer but did not observe similar associations. The Mut-High group of LUSC patients (*n* = 6) had worse outcomes than the Mut-Low group (*n* = 22), but both were likely better than *POLE*-wild patients (*n* = 469, *P* = 0.094, Additional file 2: Figure S2C and Additional file 3: Table S4). Similar to LUAD cancer, *PD-L1* expression cannot stratify *POLE*-wild LUSC patients by OS (*P =* 0.76, 95 High vs. 374 Low, Additional file 2: Figure S2D).

Tumor infiltrating lymphocytes (TILs) have been identified as a good prognostic predictor in several cancer types. For LUAD patients, *POLE* mutations were moderately associated with higher TIL percentage (*P =* 0.42, Additional file 2: Figure S2E), but not favorable outcomes (Fig. 1d). We tested whether Mut-High patients had better outcomes as a result of higher numbers of TIL. The results showed that Mut-High patients indeed contained lower TIL (*P* = 9.0e-4, Fig. 2c) compared to Mut-Low patients, but still experienced a better outcome relative to mortality rate (0/2 = 0% vs. 10/11 = 90.9% at 5.5-year follow-up), although survival curve comparison was not statistically significant probably due to the small sample size (*P =* 0.35, Additional file 2: Figure S2F). This observation suggests that *PD-L1* performs functions in *POLE*-mutant patients that are not due to the existence of TIL.

### Genes promoting tumors are mutated in Mut-high but not Mut-low patients

We compared the mutation landscapes of Mut-High and Mut-Low LUAD patients, and identified multiple genes differentially mutated between the two groups, such as *KNDC1*, *ENOX1* and *CACNA1H* (*P* < 0.05, Additional file 2: Figure S3). Gene Set Enrichment Analysis (GSEA) analysis showed that these genes are enriched in olfactory transduction that promote cancer cell invasiveness and metastasis emergence [9]. They also are involved in G-protein coupled receptor activity that stimulates cell proliferation in various cell types, and have a crucial role in many aggressive human cancers, including SCLC, pancreatic cancer, and prostate cancer [10]. The mutation of these genes may cause the loss of function and contribute to improved survivals upon treatment.

### Antitumor immune response is activated in Mut-high LUAD patients

**Fig. 3:**
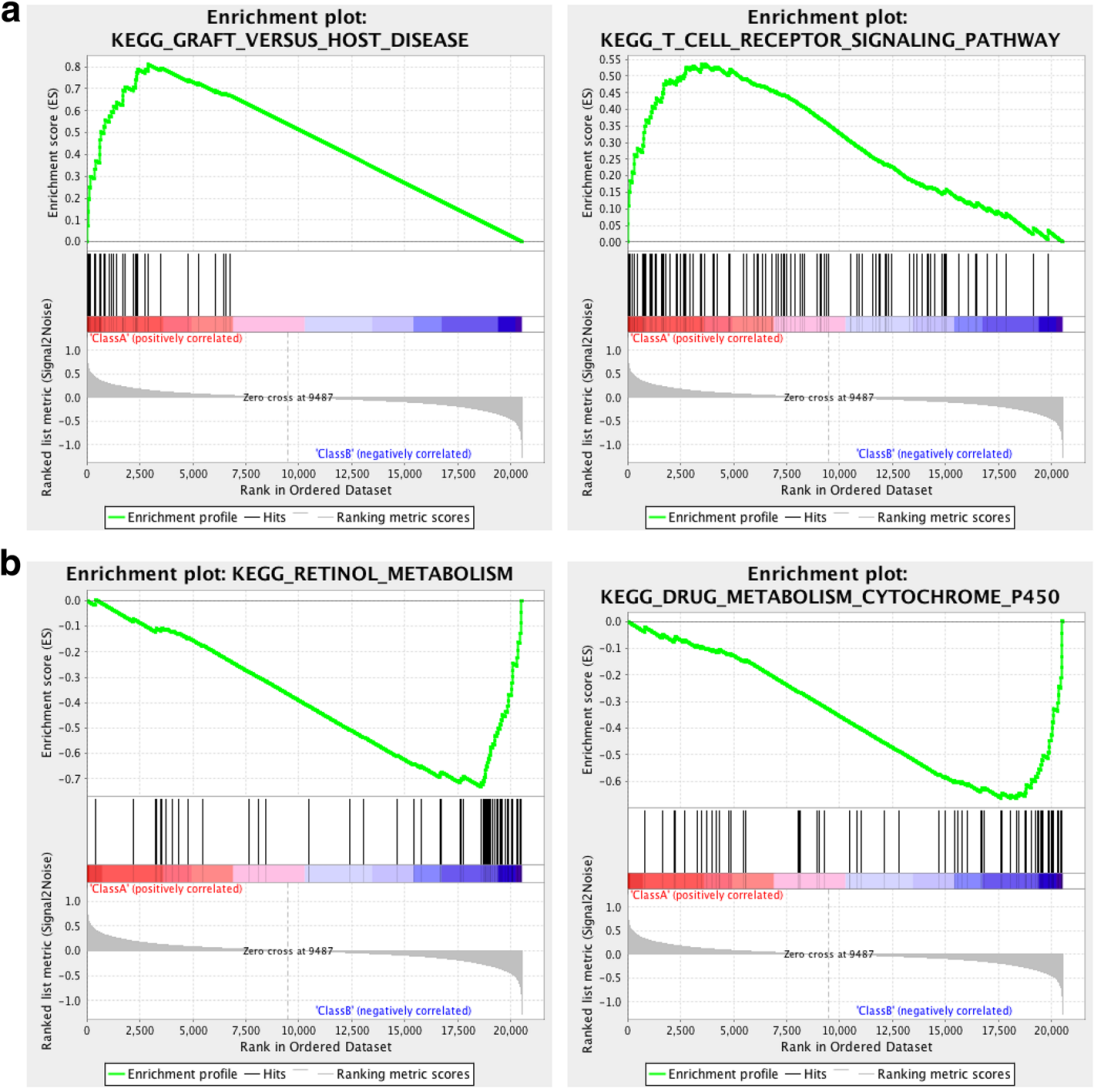
GSEA enrichment results of differentially expressed genes in the (**a**) Mut-High and (**b**) Mut-Low subgroups. ES, enrichment score

Gene expression analysis showed that signatures of immune response were upregulated in the Mut-High patients compared to the Mut-Low cases (Additional file 2: Figure S4A). Further, GSEA showed that immune related pathways, such as T cell receptor signaling, JAK-STAT signaling, and B cell receptor signaling pathways, were activated in Mut-High group (Fig. 3a and Additional file 2: Figure S4B), indicating that the immune system was activated in this group and, therefore, benefited the patient outcomes. Metabolic pathways related to retinol metabolism, and drug and xenobiotic metabolism through cytochrome p450 were activated in the Mut-Low group (Fig. 3b and Additional file 2: Figure S4B). This may suggest their poor responses to IO treatment. Similar results were achieved using the gene ontology (GO) analysis (Additional file 2: Figure S4C).

## Conclusions

LUSC and LUAD, as the two main subtypes of NSCLC, are distinct in disease pathology, smoking associations, metastatic trends, molecular mechanisms, treatment options, and patient outcomes. We demonstrated the distinct prognostic values of *POLE* mutation and *PD-L1* expression in these two subtypes. Importantly, we revealed the combination of *POLE* mutation and *PD-L1* expression as a favorable indicator for improved OS of LUAD patients and the activation of antitumor immune system. Our results identify the molecular signatures associated with *POLE* mutations and *PD-L1* expression in LUAD and LUSC and may reveal a distinct response status to chemotherapy and immunotherapy, which needs further experiments to validate.

## Additional files

Additional file 1: Methods and Materials. (DOCX 30 kb)

Additional file 2: **Figure S1.** (A) Lollipop plot shows the distribution of *POLE* mutations in UCEC, LUAD and LUSC cancers. (B) TMB cannot stratify LUAD patients. **Figure S2.** *PD-L1* expression cannot stratify (A) LUAD or (D) LUSC patients without *POLE* mutations. (B) *POLE* mutation is associated with higher mutation rates. (C) The combination of *POLE* mutations and *PD-L1* expression is not predictive to LUSC patient outcomes. (E) *POLE*-mutant patients have slighter higher percentages of TIL. (F) Mut-High group of patients have lower TIL but better survivals. **Figure S3.** There were 96 genes that were identified to be significantly mutated in Mut-High group but not Mut-Low group with *P* < 0.05 (Fisher’s exact test). **Figure S4.** (A) Comparisons of immune-related gene expression in Mut-High and Mut-Low groups. (B) GSEA pathway enrichment and (C) GO function enrichment of the differentially expressed genes in Mut-High and Mut-Low groups. (DOCX 16386 kb)

Additional file 3: **Table S1.** Descriptive characteristics of the *POLE*-mutant and -wild TCGA LUSC patients. **Table S2** Descriptive characteristics of the *POLE*-mutant and -wild TCGA LUAD patients. **Table S3.** Descriptive characteristics of the Mut-High and -Low TCGA LUAD patients. **Table S4.** Descriptive characteristics of the Mut-High and -Low TCGA LUSC patients. (XLSX 14 kb)
