# Supplementary material for: Favorable outcome of patients with lung adenocarcinoma harboring POLE mutations and expressing high PD-L1
Source: Mol Cancer. 2018 Apr 12;17:81. doi: 10.1186/s12943-018-0832-y (PMC5897927; doi:10.1186/s12943-018-0832-y)
Supplement: Supplementary file 1 — Methods and Materials. (DOCX 30 kb) [file 12943_2018_832_MOESM1_ESM.docx]

**Methods and Materials**

The TCGA Data Portal was utilized to access somatic mutations, gene expression (log2 transformed FPKM), read count, and clinical data for the LUSC, LUAD, UCEC and CRC cancers. We extracted patient-level files from the Broad Institute TCGA GDAC Firehose repository, which had been previously curated by TCGA analysis working group experts to ensure strict quality control. Data on tumor infiltrating lymphocytes (TILs) was available for a portion of LUAD patients and was downloaded, as well.

The patients were categorized differently according to study purposes. We categorized patients into *POLE*-mutant and -wild groups for the study of the prognostic values of *POLE* mutations in LUSC and LUAD. We categorized patients into high (top 20%), low (bottom 20%) and intermediate (others) groups according to *PD-L1* expression level to study the prognostic values of *PD-L1* alone. We also considered high- (top 20%) and low-TMB (bottom 20%) groups to study the predictive values of TMB in LUAD. When studying the prognostic values of the combination of *POLE* mutations and *PD-L1* expression levels, we further categorized *POLE*-mutant LUAD/LUSC patients into two groups based on their *PD-L1* expression levels: high- (top 20%, Mut-High) and low- (other 80%, Mut-Low) *PD-L1* groups.

Mutation frequency for each gene was calculated as the percentage of cases carrying at least one mutation. The comparison between the Mut-High and Mut-Low groups was performed using Fisher’s exact test.

Differentially expressed genes between the Mut-High and Mut-Low groups were identified with the DESeq2 software. The canonical pathway feature of Ingenuity Pathway Analysis (IPA, Ingenuity Systems Inc., Redwood City, CA), Gene Set Enrichment Analysis (GSEA) and GO database were used to identify the enriched pathways.

Kaplan-Meier survival analysis was performed using the R/Bioconductor package “survival.” The log-rank test, as implemented in the survival R package, was used to compare overall survivals between different groups of patients. For experiments with two groups, statistical analysis was performed using two-tailed Student’s t-test. Significance was defined as *P* < 0.05. Analyses were primarily performed using the R software.
