# Supplementary material for: Favorable outcome of patients with lung adenocarcinoma harboring POLE mutations and expressing high PD-L1
Source: Mol Cancer. 2018 Apr 12;17:81. doi: 10.1186/s12943-018-0832-y (PMC5897927; doi:10.1186/s12943-018-0832-y)
Supplement: Supplementary file 2 — Figure S1. (A) Lollipop plot shows the distribution of POLE mutations in UCEC, LUAD and LUSC cancers. (B) TMB cannot stratify LUAD patients. Figure S2. PD-L1 expression cannot stratify (A) LUAD or (D) LUSC patients without POLE mutations. (B) POLE mutation is associated with higher mutation rates. (C) The combination of POLE mutations and PD-L1 expression is not predictive to LUSC patient outcomes. (E) POLE-mutant patients have slighter higher percentages of TIL. (F) Mut-High group of patients have lower TIL but better survivals. Figure S3. There were 96 genes that were identified to be significantly mutated in Mut-High group but not Mut-Low group with P < 0.05 (Fisher’s exact test). Figure S4. (A) Comparisons of immune-related gene expression in Mut-High and Mut-Low groups. (B) GSEA pathway enrichment and (C) GO function enrichment of the differentially expressed genes in Mut-High and Mut-Low groups. (DOCX 16386 kb) [file 12943_2018_832_MOESM2_ESM.docx]

**Figure S1.** (A) Lollipop plot shows the distribution of *POLE* mutations in UCEC, LUAD and LUSC cancers. (B) TMB cannot stratify LUAD patients.

**Figure S2.** *PD-L1* expression cannot stratify (A) LUAD or (D) LUSC patients without *POLE* mutations. (B) *POLE* mutation is associated with higher mutation rates. (C) The combination of *POLE* mutations and *PD-L1* expression is not predictive to LUSC patient outcomes. (E) *POLE*-mutant patients have slighter higher percentages of TIL. (F) Mut-High group of patients have lower TIL but better survivals.


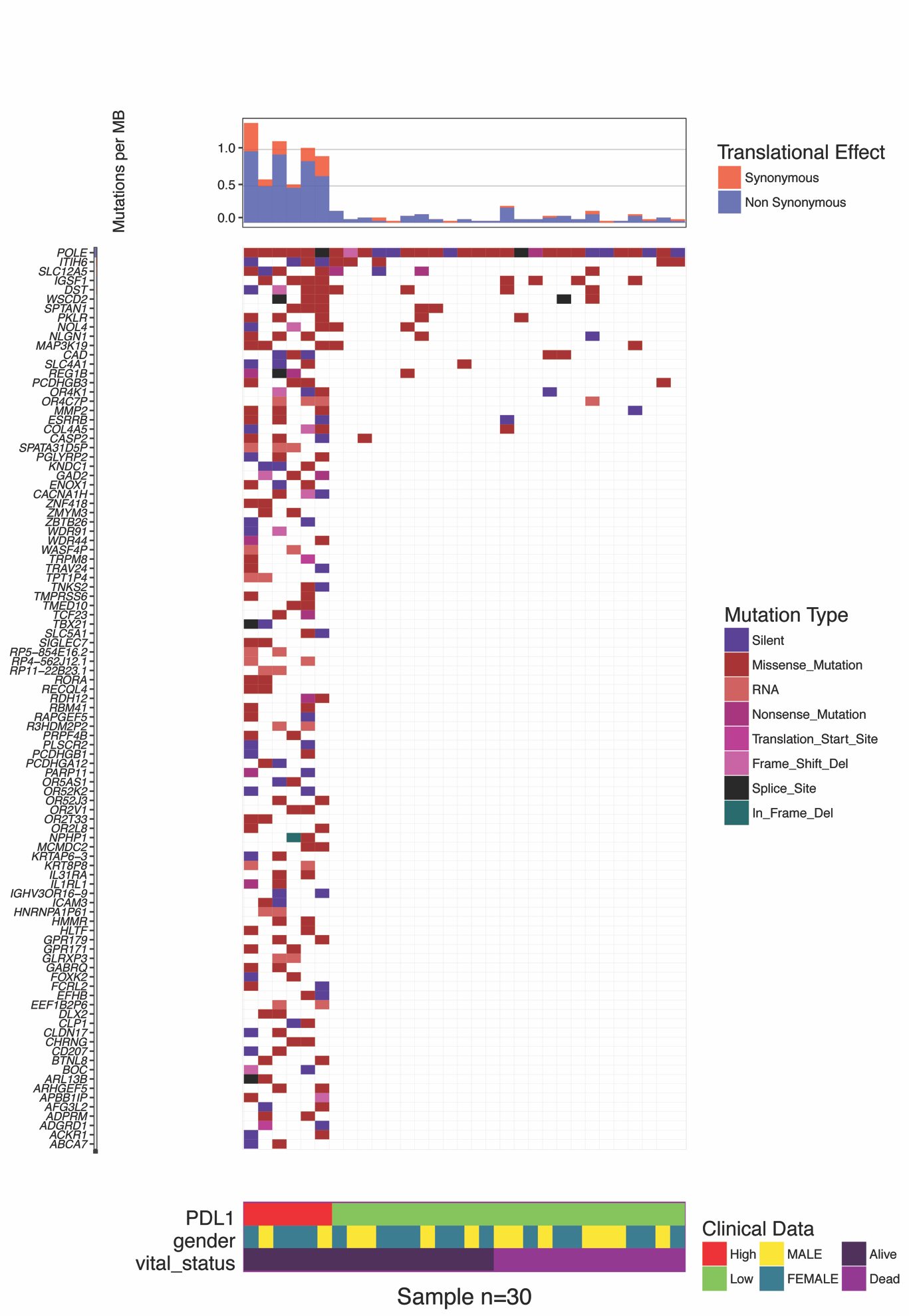


**Figure S3.** There were 96 genes that were identified to be significantly mutated in Mut-High group but not Mut-Low group with *P*<0.05 (Fisher’s exact test).

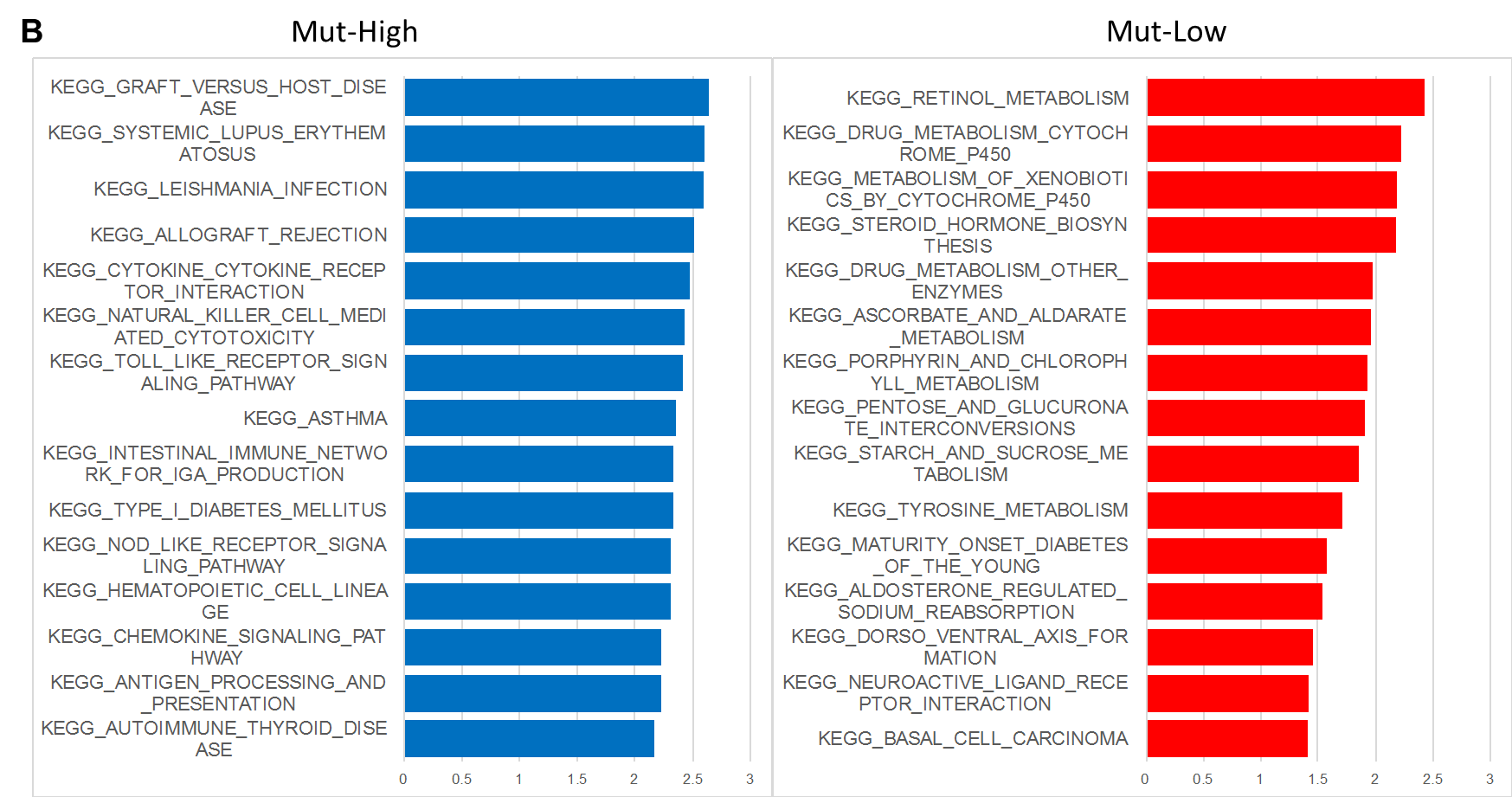


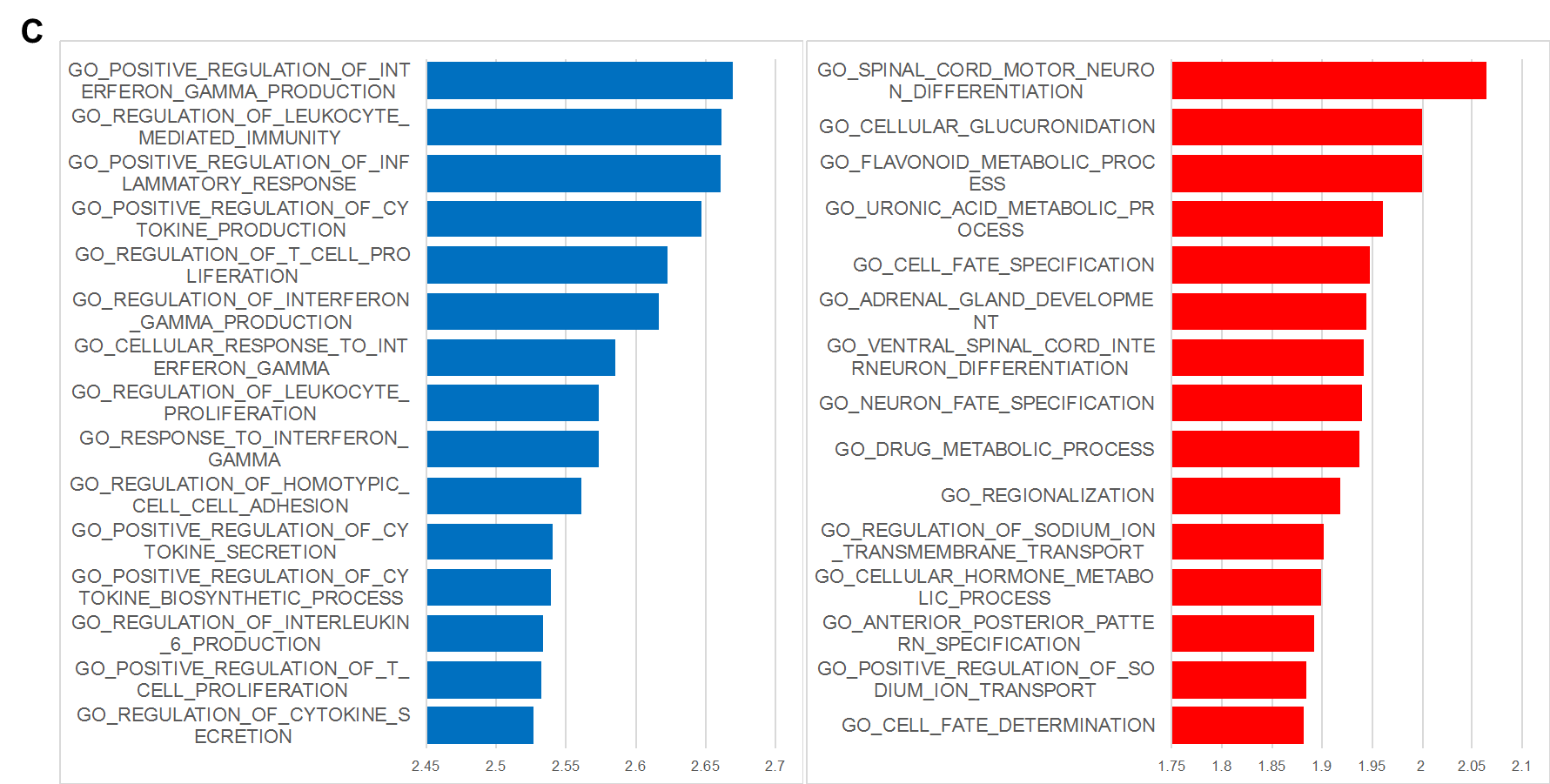


**Figure S4.** (A) Comparisons of immune-related gene expression in Mut-High and Mut-Low groups. (B) GSEA pathway enrichment and (C) GO function enrichment of the differentially expressed genes in Mut-High and Mut-Low groups**.**
